# The Lethal and Sub-Lethal Effects of Fluorinated and Copper-Based Pesticides—A Review

**DOI:** 10.3390/ijerph20043706

**Published:** 2023-02-19

**Authors:** Andreia F. Mesquita, Fernando J. M. Gonçalves, Ana M. M. Gonçalves

**Affiliations:** 1 Department of Biology and CESAM, University of Aveiro, 3810-193 Aveiro, Portugal; 2 University of Coimbra, MARE-Marine and Environmental Sciences Centre/ARNET—Aquatic Research Network, Department of Life Sciences, 3000-456 Coimbra, Portugal

**Keywords:** copper sulphate, oxyfluorfen, ecotoxicological effects, aquatic species, biomarkers

## Abstract

In recent decades, pollution levels have increased, mainly as a result of the intensive anthropogenic activities such industrial development, intensive agricultural practices, among others. The impact of metals and organic contaminants is, nowadays, a great concern to the scientific and political communities. Copper compounds are the main sold pesticides in Europe, as well as herbicides, including glyphosate. Diphenyl ethers are the second ones most sold. Glyphosate and copper compounds are intensively studied, but the opposite is seen in the case of diphenyl ethers, including fluorinated pesticides (e.g., oxyfluorfen). Some research has been performed to increase the knowledge about these contaminants, daily inputted on the aquatic systems and with dangerous effects at physical and biochemical levels on the organisms. A wide range of biomarkers (e.g., growth, survival, reproductive success, enzymatic activity, lipid metabolism) has been applied to determine the potential effects in many species. This review intends to: (a) perform a compilation of the knowledge in previous research about the action mode of organic (fluorinated-based herbicide) and inorganic (copper-based pesticides) contaminants; (b) carry out an information survey about the lethal and sub-lethal effects of the fluorinated-based pesticides, namely the oxyfluorfen and the copper-based pesticides, on aquatic species from different trophic levels, according to in vitro and in vivo studies; (c) understand the impact of oxyfluorfen and copper-based pesticides, considering their effects reported in in vitro studies and, simultaneously, the authorized concentrations by legal organizations and the effective concentrations of each pollutant found in the environment. The literature analyzed revealed noxious effects of Cu and oxyfluorfen to aquatic organisms, including freshwater and marine species, even when exposed to the reference as well as to environmental concentrations, thus highlighting the importance of more monitoring and ecotoxicological studies, to chemical pollutants and different species from different ecological niches, to sustain and improve the legislation.

## 1. Introduction

Human populations are increasing and, consequently, increasing the food production need. To suppress the production needs, pesticides and fertilizers are widely used in the agricultural practices [1], and according to the CAS (Chemical Abstract Service), there are more than eighty-nine million reported chemical compounds. Daily, an increase in the number of pollutants from anthropogenic activities on the aquatic systems is reported due to the input of novel products [2,3]. The trace concentrations together with the large number and types of pollutants represent a challenge in recognition and remediation [4]. 

According to [5], in the last 3 decades, pesticides use has increased about 1 million tons. In addition, the Eurostat agency reported 310,739 tons of sold pesticides in Europe in 2019, with the main pesticides categories being fungicides and bactericides (39.81%), and herbicides, haulm destructors, and moss killers (31.75%). Moreover, among the fungicides and bactericides, the greatest part is from inorganic origin (54%), including mainly copper compounds, as well as inorganic sulfur and other inorganic fungicides. The herbicides, haulm destructors, and moss killers category was composed mostly of ‘Other herbicides’ (61.8%), including glyphosate (a well-studied pesticide) and fluorinated herbicides [6], a group with an increasing application, as it is seen as an alternative to control glyphosate-resistant weeds [7]. 

Fluorinated pesticides may act by different modes on the organisms: (a) inhibition of the acetolactate synthase, an enzyme with an important role on the synthesis of ramified chain amino acids [8,9]; (b) inhibition of Acetil-CoA Carboxilase, which catalyzes the first step of the fatty acids’ biosynthesis on plants [10,11]; (c) inhibition of mitosis and consequent cell division [10]; (d) inhibition of protoporphyrinogen oxidase or ‘Protox’, a key enzyme that catalyzes the oxidation of protoporphyrinogen IX into protoporphyrin IX, essential on the chlorophyll biosynthetic pathway [10,12,13]; (e) inhibition of 4-hydroxyphenylpiruvate dioxygenase, an enzyme involved in the tyrosine biosynthesis, affecting the chlorophyll production [14,15]; (f) inhibition of very long-chain fatty acids elongase, with effects on the biosynthesis of these fatty acids [16,17,18]. According to the different action modes of these pesticides, detrimental effects such as inhibition of photosynthesis and synthesis of photosynthetic pigments, amino acids synthesis inhibition, and changes in the fatty acids and protein metabolism have been reported [19,20,21,22,23]. Thus, the fatty acids and amino acids determination, as well as the assessment of lipid peroxidation and photosynthetic pigments, may be valuable biomarkers on the monitoring and health status of the aquatic organisms.

Some researchers have also been dedicated to understanding the copper action mode. Currently, three processes have been reported that may explain the mode of action of copper ions—(a) inactivation of proteins due to the interactions between copper (II) and thiol-, imidazole-, and carboxyl-groups of amino acids; (b) formation of copper (I), through the interactions between copper (II) and deoxidants; the copper (I) may act as a catalyzation agent on the formation of hydroxyl radicals, a reactive oxygen species (ROS); (c) copper (II) and copper (I) may replace essential cations from specific binding sites [24,25]. Consequently, several detrimental effects on the photosynthesis process, fatty acids metabolism, carbohydrates synthesis [18,26,27,28,29], cell respiration, ATP production, pigments synthesis, and inhibition of cell division [30] have been associated with the exposure at high copper concentrations, with consequences on the function and structure of the cell membrane, loss of key nutrients, and metabolic imbalance [31].

UNESCO reported an input of pollutants into the aquatic systems of 730 million tons per year [32]. Marine systems have a key role in human life, as these systems provide food sources and natural resources with application in different sectors such as pharmaceutical, cosmetic, agriculture and animal feeding, and recreational and professional activities. However, these systems are often under anthropogenic pressures such as sewage water discharges, input of contaminants from industrial and agricultural activities, and plastic pollution. These continuous anthropogenic pressures affect the water quality and, thus, the aquatic communities [33].

First, changes in the structure or function of microbial communities, which is very sensitive to the changes in the water quality, may affect the entire aquatic ecosystem [34]. The quality and abundance of bacterial communities, and micro- and macroalgae depend on the environmental conditions [35]. These organisms are at the base of the trophic chain acting as primary producers, presenting a key role in the trophic chain. Zooplankton acts like links between primary producers and secondary consumers. The exposure to stressors may affect its nutritive value, with effects on the lipid metabolism [24]. In the case of bivalves, which are greatly appreciated as a food source by human beings, chemical stressors may affect greatly their behavioral (feeding, growth, reproduction, cardiac activity, and maturation) [36] and the biochemical pathways (decrease in the ATP; lipid peroxidation and cell glutathione imbalance; inhibition of respiratory process; changes in the enzymatic activity, proteins synthase, and lipids profile) [37]. Fish are the first source of highly unsaturated fatty acids to the human population. These organisms are greatly affected by metals, such as copper, and organic contaminants, such as fluorinated pesticides, with consequences on the enzymatic activity, oxidative stress, and changes in the lipid metabolism and fatty acids profile with a decrease in its nutritive value (decrease in omega-3 and -6, and increase in saturated fatty acids) [27]. Considering the increased production and input of the pollutants into the environment, as well as the several adverse effects on the ecosystems and communities, it becomes very important to monitor and to diagnose the ecosystems exposure to these organic and inorganic pollutants by applying biomarker tools. 

According to the studies conducted thus far about both chemicals, it is possible to verify that the exposure to these compounds comprises effects on the behavior (e.g., with alteration in the growth), lethal [18,20,29,38,39,40,41,42,43,44], and biochemical levels (e.g., with changes in the enzymatic activities, lipid metabolism, and proteins expression) [18,19,20,21,22,23,29,41,44,45,46,47,48,49,50,51,52,53,54]. 

Metals and organic pollutants have been reported as inductors of reactive oxygen species (ROS) formation [55,56]. ROS are the group of superoxide, hydroxyl, and non-radical oxygen derivatives (e.g., hydrogen peroxide and singlet oxygen) [57]. When the antioxidant system does not act efficiently against the ROS action, the organisms suffer oxidative stress caused by biological, chemical, and physical stress [58]. Thus, to fight the oxidative stress, aerobic cells have developed antioxidant defense systems or a redox balance [59]. Among several mechanisms and agents of antioxidant defense, metallothioneins (MTs), catalase (CAT), glutathione peroxidase (GPx), glutathione *S*-transferase (GST), superoxide dismutase (SOD), species reactive of thiobarbituric acid (TBARS), and cholinesterase (ChE) are mostly the biomarkers used in the environmental assessment [60,61,62]. Moreover, reduced glutathione (GSH) may act as a substrate of GST and also takes part in the xenobiotics conjugation and detoxification [61]. Lactate dehydrogenase (LDH) plays a key role in the anaerobic metabolism [63,64]. ROS action, when it is non-controlled, may have deleterious effects on pigments, proteins, lipids, DNA, RNA, ribosomal synthesis, and enzymatic systems [65,66]. The induction of enzymatic or non-enzymatic antioxidant defenses can provide relevant information on the study of the effects of chemical compounds on many organisms [67]. Thus, biomarkers may be defined as powerful tools and endpoints in toxicological studies to assess the organisms’ health status and consequently to detect changes and stress conditions on the ecosystems.

Fatty acids (FAs) and carbohydrates have also been used as biomarkers to assess the organisms’ health status [24,68] and exposure to stress conditions, such as organic and inorganic pollution, by copper and fluorinated pesticides [18,22,29,35,41,45,52]. 

The determination of changes in the fatty acids profile, such as inhibition of elongase of very long-chain fatty acids induced by fluorinated-based pesticides, or effects on the esterification, desaturation, and mobilization of triacylglycerols associated with copper exposure [69], is revealed to be an important point in the assessment of the aquatic organisms health status, as fatty acids are essential components in the cell membrane with crucial physiological functions (e.g., permeability). Moreover, FAs are one of the main lipid constituents of the cell membranes and are used as fuel for the metabolic processes at all trophic levels [70]. Furthermore, FAs are the most important molecules transferred along the trophic chain (from primary producers to consumers) and, as the animals are not able to synthetize some of them, the so-called essential FAs (EFAs) are essentially acquired by food sources. Thus, FAs have been used as trophic markers [29,71,72,73] and as qualitative markers allowing for determining predator–prey relationships [74,75,76]. 

Some researchers have also reported effects on carbohydrates composition to different organisms, because of copper and fluorinated pesticides exposure. However, the response depends on the organisms. There are studies that report a decrease in carbohydrates to *Eisenia foetida* [77] and to *Chaetoceros calcitrans* and *Nitzchia closterium* [78], as a consequence of the exposure to low concentrations of a fluorinated herbicide (oxyfluorfen). Other studies highlight an increase in the glucose levels on *Thalassiosira weissflogii* after exposure to copper and oxyfluorfen [18] and the accumulation of non-structural carbohydrates, at high levels of copper on *Citrus grandis* and *Citrus sinensis* [79]. Carbohydrates have a key role in vital functions, such as in cell homeostasis maintenance in the immunity defense process. In addition, they are the main and speedy energy source to deal with stress conditions. However, pollutants may cause great energetic costs to the immunity balance [80]. Moreover, changes in these biomolecules content or storage may affect several cell functions, such as carbon fixation, lipid accumulation, or photosynthetic efficiency [81]. Polysaccharides are carbohydrate polymers composed of long-chain monosaccharides and they have been reported as protectors of the bacterial cells against metals, organic compounds, and many other environmental perturbations [82,83,84,85]. Therefore, this review aims to: (a) perform a collection of the literature about the action mode of organic (fluorinated herbicide) and inorganic (copper pesticides) contaminants; (b) assess the relevance of the lethal and sub-lethal effects of the fluorinated-based pesticides, namely the oxyfluorfen and the copper-based pesticides, on aquatic species from different trophic levels, reported by in vitro studies, but considering the existent environmental data; (c) understand the danger of oxyfluorfen and copper-based pesticides to aquatic systems, considering their effects reported in in vitro studies and, simultaneously, the authorized concentrations by legal identities and the concentrations of each pollutant found in the environment. Moreover, this review highlights the toxicological and biochemical effects as a consequence of the fluorinated and copper-based pesticides already studied in several species along the trophic food chain and ecological niches. It also emphasizes the harmful effects of these pollutants on the ecosystems, once concentrations reported in the environment are higher than those used in in vitro bioassays, which have had dangerous effects on freshwater and marine organisms, such as algae, zooplankton, macroinvertebrates, and fish species.

## 2. Methodology

This review article performs a compilation of the information from 236 sources. The literature review was based on the main keywords: pesticides, herbicides, organic pollutants, inorganic pollutants, metals, fluorinated based herbicides, oxyfluorfen, copper, aquatic organisms, toxicological effects. Some research platforms were used, namely Google, Google scholar, B-on, Science Direct, Pesticides Data Base, European Commission site, the USEPA site, and the World Health Organization site. Moreover, a literature search for privileged recent works from 2000 to 2023 was performed; however, some knowledge bases crucial to this work clarification required the use of older sources, namely studies since 1981. 

## 3. Organic Contaminants—A General Characterization

Many works have reported the organic contaminants as an increasing concern given its deleterious effects on the environment [86,87,88]. Numerous organic contaminants are reported as hardly biodegraded and lipophilic compounds, therefore, have a great bioaccumulation ability [86,89,90]. Moreover, these contaminants are described as mutagenic, carcinogenic, endocrine disruptors, immune system depressors, reproductive impairment originators, hyperthyroidism causers, neurotoxic, and responsible for changes in skeletal growth and ontogenetic development [86,89,90,91].

Organic pollutants include phenolic compounds, agrochemicals (organic pesticides and herbicides), industrial chemicals (e.g., polychlorinated biphenyls—PCBs), and products from industrial processes (e.g., polychlorinated dibenzo-p-dioxins—PCDDs, polychlorinated dibenzofurans—PCDFs, and polycyclic aromatic hydrocarbons—PAHs) [86,87,88,92]. Many of these compounds are persistent, designated as persistent organic pollutants (POPs). POPs are chemical substances that, due to its persistence, may bioaccumulate through the trophic chain and be carried and reach other systems far from its source, with consequences on the environmental and public health at the global level, even in places that have never produced or been used [27]. 

### 3.1. Pesticides

Pesticides comprise a large spread of environmental contamination. The human population is increasing exponentially, and the fertilizers and pesticides used have become essential to the agriculture practices, in order to suppress the food production needs. However, the excessive use of these compounds may comprise deleterious effects to the ecosystems (e.g., biodiversity loss and damage to public health) [93,94] and has become unescapable on freshwater and marine ecosystems [95]. Some studies about pesticides have been developed, as one of the main harms of this chemicals is its non-selectivity, disturbing non-target organisms and consequently the normal function and maintenance of the terrestrial and aquatic systems [96]. Moreover, when applied in soils and plants and depending on the persistence on the environment, the pesticides are subjected to many processes that may involve degradation and/or conveyance by drift, leaching, and run off to several compartments (soil, water, sediment) [97]. 

According to the World Health Organization (WHO), pesticides are defined as chemical compounds whose main aim is to kill pests, including insects, fungi, rodents, and weeds [98]. Nonetheless, according to the Environmental Protect Agency of the United States (USEPA), pesticides are any substance used to prevent, destroy, repel, or mitigate any pests, with pests being characterized as any animal, plant, or microorganism that affects human food, health, or wellness [99].

Pesticides may be classified according to its target (herbicides, fungicides, insecticides, repellents, avicides, acaricides, bactericides, viricides, etc.), chemical composition of the active ingredients (carbamates, organochlorine, organophosphorus, pyretrine, pyretroids, etc.), and according to the risk (extreme danger, high danger, moderate danger, and slight danger). The exposure pathways may be by ingestion (of contaminated water and food), inhalation (of contaminated water, dust, soil, and industrial vapors), and dermal contact [100]. 

Several pesticides are recognized as endocrine disruptors—exogenous substances (natural or synthetic) that affect the endocrine system and may cause damage to the physiological pathways of the organism and/or on the next generations, with deleterious effects on the development, growth, and reproduction. As the transcriptional activity of nuclear receptors is one the first targets of the endocrine disruptors, many pesticides (organochlorine, diphenyl ethers, organophosphorus, carbamates, acid amines, ureas, pyretroids…) were designed to act against these nuclear receptors [101]. 

Pesticides exposure is associated with some diseases in humans such as Hodgkin’s disease, non-Hodgkin lymphoma [102,103], Parkinson’s [104,105], endocrine disruption [106,107], breath and reproductive problems [108], lung cancer [109], and brain tumors [110]; damage to the lymphatic tissues, liver, thyroid, uterus, and mammary gland (according to animal tests of low concentrations exposure—0.1 ppm of Dieldrin) [111]; increased risk of prostate cancer [112].

### 3.2. Herbicides—A Main Concern

Herbicides are the most used pesticides, responsible for 40% of the world’s pesticides production [113], followed by insecticides, fungicides, and other pesticides [99]. One of the greatest apprehensions about the contamination by herbicides is due to its ability of bioaccumulation on primary producers and consequent proliferation along of the trophic chain [114]. 

According to the Weed Science Society of America (WSSA), the herbicides may be classified into 28 groups consistent with their action mode, which often includes target metabolic enzymes, carriers, hormonal regulators, cofactors, proteins, or other cell biomolecules [115]. The herbicides action mode has a key role on its effectiveness. Furthermore, the damage severity and quickness are linked to the herbicide power against the target, as well as the target biological relevance [116].

#### 3.2.1. Fluorinated-based herbicides

Fluorinated organic products are present mostly on the agrochemical products [117,118,119]. About 25% (56/229) of compounds have at least one fluorine atom, often present as substituents aryl-F, aryl-CF3, and aryl-OCF3 [120]. In organic chemistry, the C-F bonds are the strongest and fluorine is able to bind covalently to the next smallest carbon atom. Therefore, the change from F to H has a negligible steric perturbation and leads to a stable derivative. The polarity linked to C-F bonds [121,122] will bring dipoles with consequent changes to the conformation, leading to a better bonds target. Moreover, the incorporation of fluorine may modify the surrounding protic functional groups acidity, namely of the groups OH, NRH, and CO2H. Thus, considering that many fluorine substituents usually increase the lipophilicity and the molecules bioavailability to cross the cell membranes, and specific sites of the fluorine or fluorinated groups may be used in the protection against or suppress in vivo metabolism, fluorine may comprise several effects such as inhibition of biological processes (e.g., cell division, fatty acids biosynthesis including very long-chain fatty acids), inhibition of chlorophyll production, or further degradation, leading to leaf bleaching or generation of oxygen singlets and overproduction of oxygen reactive species, resulting in lipid peroxidation, which supports the development of compounds with a great efficacy [1].

The fluorinated-based herbicides can be classified according to its action mode, as different herbicides may act on different targets, as described in Table 1. 

**Table 1:** Fluorinated-based herbicides and its action modes.

Inhibitors Type	Target Characterization	Action Mode	Chemical Compounds	Aim/Application
Acetolactate Synthase (ALS) Inhibitor	ALS is a flavin enzyme involved in the biosynthesis of the ramified chain of the amino acids L-valine, L-leucine, and L-isoleucine [10,15]	Act over the ALS, leading to the decrease in the synthesis of the amino-acids-ramified chain, essential to the early tissues growth [8,9]	Sulphonamides; Flumetsulam; Florasulam; Penoxsulam; Piroxsulam.	Fight against broadleaf weeds on wheat crops and other cereals [123]
Acetil-CoA Carboxylase (ACC) Inhibitors	ACC catalyzes the 1st step of the fatty acids’ biosynthesis on plants. ACC catalyzes the conversion from Acetil-CoA to Malonil-CoA—with a key role on the saturated fatty acids assembly in the plastids.	ACC inhibition prevents the fatty acids biosynthesis and drains the Malonil-CoA levels in the cell to the additional elongation of SFAs, when they are transported from the plastids to the cytosol [10,11]	Clodinafop-propargil; Fluazifop; Fluazifop-P-butyl; Haloxifop.	Cereal crops
Mitosis Inhibitors	Cell division	Chemicals enter the cell and establish a link with tubulin, causing disruption to the microtubules formation and, therefore, inhibition of mitosis [10]	Trifluralin; Ethalfluralin.	Prevent the growth of weeds on the crops
Synthetic Auxins	Plant hormones	Auxins are applied and adsorbed by the leaf and quickly distributed through the plant. They induce auxins as a response, leading to the atypical development of morphologies [124].	Fluoroxipir	
	PDSs are involved in the carotenoids biosynthesis; Catalyzes the conversion from phytoene to carotene and phytofluene in plants	PDS inhibition causes chlorophyll degradation and consequent bleaching of the leaves [125,126]	Diflufenican	
Protoporphyrinogen Oxidase (PPO) or ‘Protox’ Inhibitors	PPO is a key enzyme that catalyzes the oxidation of protoporphyrinogen IX into protoporphyrin IX, an intermediate essential to the chlorophyll biosynthetic pathway [10,12] and to the chloroplasts, and then it is distributed through the chloroplast membrane [127]	PPO inhibitors block the protoporphyrin production; Accumulation of protoporphyrinogen on the chloroplasts; Leakage of protoporphyrinogen to the cytosol and non-enzymatic oxidation; Massive production of singlet oxygen and lipid peroxidation [13]	Oxifluorfen; Fomesafen; Lactofen.	
4-Hydroxyphenylpiruvate dioxygenase (HPPD)Inhibitors	HPPD is an enzyme involved on the tyrosine biosynthesis; HPPD converts tyrosine into homogentisate, a metabolic precursor to plastoquinone and tocopherol in plants.	HPPD inhibitors affects the chlorophyll production, causing bleaching [14,15]	Isoxaflutole; Pyrasulfatole; Tembotrione.	Fight against broadleaf and grass weeds on rice and maize farms.
Very Long-Chain Fatty Acids (VLCFAs) Elongase Inhibitors	VLCFAs have an essential importance in the formation of glycosylphosphatidyl-inositol anchors and sphingolipids [128].	Inhibition of the elongase that catalyzes the very long-chain fatty acids biosynthesis [16,17]	Flufenacet.	Prevent weeds on cereals and corn crops.

#### 3.2.2. Oxyfluorfen and Its Effects

Oxyfluorfen was introduced by Dow in 1976 and has been widely used in rice crops due to its high efficiency as a herbicide and low application range. However, the intensive use may affect environmental and human health [129]. The oxyfluorfen application has increased on pre-emergence and early post-emergence, as, nowadays, this non-selectivity and the broad-spectrum herbicide are seen as an alternative to control the glyphosate-resistant weeds; however, the decrease in oxyfluorfen efficiency is also expected [7]. According to ECHA [130], maximum limits of oxyfluorfen in different food types are defined from 0.05 mg L^−1^ to 0.1 mg L^−1^; however, environmental studies have reported higher environmental concentrations, up to 23.6 mg L^−1^ in Nile River, Egypt [131].

This diphenyl ester, such as all herbicides of fast performance that act by inhibition of PPO activity, belong to group 14 of the WSSA [116]. The PPO activity inhibition and consequent accumulation of protoporphyrinogen in the chloroplasts with leakage to the cytosol, which is non-enzymatically oxidized in protoporphyrin, lead to a massive production of singlet oxygen (Table 1), and hydrogen of the unsaturated lipids is extracted; then, lipid radicals are produced, and a chain reaction of lipid peroxidation begins. Proteins and lipids are oxidized with peroxidation damage on the cell membrane and membrane permeability disruption, causing fast disintegration of organelles and cells and, in the last state, cell death [132,133,134,135,136,137,138].

According to our knowledge, few studies have been dedicated to the evaluation of oxyfluorfen’s effects on the different species, so there is a lack of information in this field mainly in marine systems. Among oxyfluorfen’s known effects, there are the inhibition of photosynthetic pigments, photosynthetic electrons transport, acetylcholinesterase activity, growth, and phosphate and nitrogen fixation ability; induction of superoxide radicals and hydrogen peroxide levels, and activity of antioxidant enzymes and the stress protein Hsp 70; changes in fatty acid profiles with the increase in saturated and monounsaturated fatty acids content. Table 2 shows a compilation of the effects already reported in some studies to freshwater organisms, such as inhibition of photosynthetic pigments and activity and growth rate in microalgae species, as well as changes in enzymatic biomarkers response in microalgae and fish species, and changes in the nutritive value of fish species [19,20,21,22,23]. Moreover, considering the great oxyfluorfen concentrations above reported for aquatic systems, the concentrations used in the in vitro studies and the observed effects represent a major concern, with real impacts to the aquatic organisms and consequently to the ecosystems function and structure.

## 4. Metal Contamination 

Metals can have several sources; they may be from anthropogenic activities such as industrial and miner activity, metal plating activities, rainwater runoff, and sewage [139,140,141], or by other sources as metals such as copper, cadmium, zinc, and lead may also be released by natural sources [43]. Moreover, metals may be divided into two categories—essential and non-essential elements. Essential elements, such as copper, iron, magnesium, and zinc, have a biological function to the organisms [142,143] but may become toxic at great concentrations [144]. On the other hand, non-essential elements (e.g., mercury, lead, cadmium, and arsenic) have no role on the metabolic processes [145,146], being toxic even at very low concentrations [144]. 

Although some metals have a key role on the normal organisms’ metabolic maintenance, at excessive concentrations, all are toxic [147]. Despite metals being naturally present in the environment, human activities have contributed to the increase in its concentrations [148]. Furthermore, high levels of CO_2_ and low pH may lead to the increase in metals solubility or to the changes in their speciation, therefore contributing to the increase in metals bioavailability [149,150,151,152]. Additionally, great levels of CO_2_ may influence the accumulation and intracellular link between metals and marine organisms [153,154,155]. Moreover, the metal concentrations may fluctuate seasonally due to the runoff variations and also according to the tidal and deep cycles; spatially according to the salinity, organic matter, temperature, and availability of other contaminants [156]; as a function of the life-cycle stage and physicochemical form of the metal [157].

Metals are persistent pollutants and exhibit hard degradation [158]. Aquatic organisms are permanently exposed to its action, as metals are present in the water column and sediments. Thus, when the metals enter in the system, they may affect, directly, the organisms by direct pathways with cell proteins, resulting in non-specific bonds, enzymatic co-factors, or transcription factors replacement and discharging of stored metals. On the other hand, the metals are able to affect indirectly the organisms by oxidative damage on the proteins or other key cell constituents [155,159,160,161,162,163]. Metals are also present in many chemical formulations, such as pesticides, with the ability to accumulate on the organisms by different processes (e.g., bioaccumulation, bioconcentration, proliferation along of the food web) [164]. The level of metals on the organisms may be considerably greater than on the environment [165], with adverse effects at biochemical and physiological pathways for the organisms, due to the overproduction of oxygen radicals, which will affect cellular processes through the oxidative damage caused by these radicals, and leading, for example, to lipid peroxidation occurrence and damage to biological membranes [166], affecting its properties, namely the permeability and fluidity [167]. In the ultimate analysis, metals may have dangerous effects on human health, as human beings by consuming these organisms may accumulate the metals present on this food resource [168]. Some authors have reported damage to organs such as the brain, liver, and kidney, with the excessive exposure to copper [169]. Cardiovascular health problems, cancer, and negative impacts on renal tubular function to the reabsorption of amino acids, proteins, and sugars, with the exposure to cadmium [170] and embryo toxicity, allergic reactions, and dermatitis [171] were also related to the exposure to copper.

Metals toxicity is often related to an increase in the free radicals’ levels that can interact with biomolecules such as DNA bases, lipases, and protein thiols, as well as to genotoxicity [172,173] and biological homeostasis disruption [174]. Therefore, metals are able to compromise growth, development, reproductive success, and survival of the organisms and consequently have an impact on the structure and function of the populations and communities to which they belong [144,166,175,176,177,178]. Thus, the indiscriminate use of metals comprises a marked environmental risk [179] with effects on the ecosystems and their biota.

### 4.1. Copper—From Essential to a Toxic Element

Copper is known as an essential element, with a key role on several biological functions such as mitochondrial and cell respiration, neurotransmitter biosynthesis, and free radical detoxification [180]. It is a cofactor of many enzymes (e.g., Cu/Zn superoxide dismutase (SOD), cytochrome c oxidase, amino oxidase, laccase, plastosyanin, polyphenol oxidase) [181,182] and the copper deficiency may result in a decrease in the enzymatic activity involved in the antioxidant defense system and, consequently, in an increase in reactive oxygen species (ROS) [183,184]. This metal also participates in hemoglobin level regulation and embryonic development [180]. Despite the key work of this micronutrient, at excessive concentrations, copper may become toxic to the organisms, as reported by several researchers. 

Excessive metal concentrations are toxic to the organisms and the copper is not an exception. Several studies have shown that copper toxicity is dependent on its speciation, with the Cu+ being the most bioavailable, so the most toxic ions [185] and the content of free ions are dependent on the physicochemical properties of the water as well as of the organic ligands’ bioavailability [186,187].

Copper is reported as a promotor of oxidative stress, as it catalyzes ROS production, when at excessive concentrations, by the generation of oxidant radicals [55,188,189], with consequences on membrane lipids and, consequently, with cell damage [56]. Similarly, this metal has been described with inhibitory effects on the acetylcholinesterase activity [190] and on the phagocytic function, by stimulation or inhibition of the ROS production, according to the concentration [191]. Moreover, copper exposure has revealed impacts at the biochemical level, with alterations on the fatty acids profile, namely the increase in saturated fatty acids and the decrease in unsaturated fatty acids content, including the decrease in essential fatty acids such as EPA. These molecules are a key structural constituent of the cell membrane phospholipid bilayer (whose variations may affect the membrane properties such as the fluidity and density); of marine species from different trophic levels, such as the diatom *Thalassiosira weissfloggi*, the zooplanktonic species *Acartia tonsa* and *Artemia franciscana*, and the bivalves *Cerastoderma edule* and *Scrobicularia plana* [18,27,29]; of the enzymatic activity, with the inhibition of digestive gland hexokinase activity associated with a decrease in GSH content on *Mytilus galloprovincialis* [192]. De Almeida et al. [193] observed an inhibition of GPx activity on the bivalves *Perna perna*, also associated with a decrease in GSH levels and with consequent increase in lipid peroxidation occurrence. Doyotte et al. [194] also reported a decrease in GSH content on *Unio tumidus* when exposed to copper. Maria and Bebianno [195] tested the effects of copper exposure on *Mytilus galloprovincialis* and observed different responses according to the analyzed tissue, verifying the activation of antioxidant enzymes, namely glutathione reductase, glutathione *S*-transferase, glutathione peroxidase, catalase, and metallothionines on gills and the inhibition on the digestive gland; only SOD activity was inhibited on both tissues. Mesquita et al. [42] evaluated the effects of copper exposure on the antioxidant defense system of two bivalves species (*Cerastoderma edule* and *Scrobicularia plana*) considering two different size classes, namely large and small organisms. In this study, an increase in the GST and GR activity at the lowest concentration was observed, with a return to the basal activity on the remaining concentrations and a gradual increase in GPx activity across the range of concentrations, to the large organisms of both species. In the case of the small organisms, an opposite trend was observed with *C. edule* and *S. plana* exhibiting the inhibition of GPx activity, keeping the activities of the remaining enzymes relatively constant. Moreover, effects of copper exposure have also been reported at the physiological level, such as the reduction in the abundance, growth inhibition, and no reproductive success of the marine organisms [196,197]. Moreover, copper has showed effects on the macrofouling invertebrates’ assemblages [198], as it is a main contaminant of antifouling paints formulations [199]. 

### 4.2. Copper Applications and Copper Sulfate Effects on Aquatic Organisms

Copper is a metal with ecological relevance and is involved in coastal pollution processes. This metal can be employed in several ways, for example, as pentahydrate copper (II) sulphate, copper (II) sulphate, dihydrate copper (II) chloride, copper (II) chloride, copper oxide, or copper (II) [27]. Copper-derived compounds are used in formulations of fungicides, bactericides, herbicides, and anti-vegetative paints [48,196,200,201,202,203,204,205,206,207,208,209,210,211,212,213,214,215,216,217,218,219]. Moreover, the use of copper nanoparticles has increased in several products, namely agrochemicals formulations, paints, antimicrobial products, catalyzers, semiconductors compounds, and sensors, with a consequent increase in the copper release to the environment, and in the terrestrial and in the aquatic systems [220,221,222,223,224]. As this metal has many applications, industrial discharges [196], urban runoff, sewers discharges [225], and antifouling biocides [226,227] are most often the contributors of copper pollution [199].

The WHO [228] defined a reference value of 2.0 mg L^−1^ to Cu; however, the same identity described the occurrence of higher values until 30 mg L^−1^ in drinking water. Moreover, environmental studies have reported Cu concentrations of about 10 mg L^−1^ in aquatic systems near urban areas, achieving 100 mg L^−1^ in aquatic bodies surrounding mining areas [229].

Copper sulphate is a chemical-based copper used in industrial and agricultural activities as an ingredient in pesticides formulations (bactericides, herbicides, fungicides, etc.). Several studies have reported the effects of this chemical at different levels, such as reproductive impairments, reduced growth, or behavioral changes [38,39,40,43,230]. Moreover, copper can lead to physiological changes and proteins dysfunction [47,230]. In fish, excessive exposure to copper may cause dangerous effects on gills, guts, and the sensorial system [40]. Effects reported in in vivo studies should alert people to the Cu toxicity, mainly because the tested concentrations are lower or similar to the reference value established for Cu and significantly lower than the concentrations found in the aquatic systems. Thus, a compilation of copper effects can be analyzed in Table 3. 

## 5. Conclusions

The utilization of chemical compounds has increased in recent decades mainly related to anthropogenic activities, becoming crucial to monitoring their impacts on the ecosystems and in the communities. This review highlights the dangerous effects of organic and inorganic pollutants, with a focus on two pesticides widely used in the world: the organic herbicide—oxyfluorfen, and the inorganic pesticide—copper sulphate. Despite the different action modes of these two chemicals, both are reported as dangerous to non-target species, and its application has increased to provide well-being and development of human activities. After an intensive analysis about the known effects of these two chemicals, reported in many studies, this review highlights a higher sensitivity of the primary producers to copper and oxyfluorfen, when compared to the consumers. Oxyfluorfen is shown to be more dangerous than copper to the organisms from different trophic levels. 

Despite the reference levels established to Cu (2.0 mg L^−1^) and oxyfluorfen (from 0.05 mg Kg^−1^ to 0.1 mg Kg^−1^ in food wells), the highest concentrations have been reported in environmental systems (up to 100 mg L^−1^ to Cu, and up to 26.3 mg L^−1^ to oxyfluorfen). Moreover, according to the in vitro studies, both reference and environmental concentrations to each contaminant can comprise a harmful effect to aquatic organisms; namely, the induction of ROS production, with changes in the antioxidant system defense; the occurrence of lipid peroxidation, with consequent changes to the fatty acids profile; the changes to the photosynthetic pathway, with a decrease in the photosynthetic pigments concentrations and in the photosynthetic carriers, are the most reported consequences of the exposure to both pesticides, with the inhibition of the acetylcholinesterase activity also having an effect often reported but associated with the oxyfluorfen exposure. Then, these impacts at the organisms’ level should lead to negative impacts on the structure and function of the ecosystems. 

This review also emphasizes the lack of information regarding the oxyfluorfen consequences on marine systems and on non-target species, with further research about this chemical at the biological level being of extreme importance, considering the known effects, as its usage is increasing daily, as well as its discharges on the aquatic systems with dangerous effects on these ecosystems and communities. It is imperative to monitor programs to aquatic systems and to assess the effects of contaminants considering lethal and sub-lethal effects, namely in terms of reproduction, growth rate, nutritive value, antioxidant defense, or neurotoxic effects, and based on these assessments to implement mitigation plans, improve the legislation, and work on the restoration of the ecosystems’ health status.

## Figures and Tables

**Table 2:** Effects of the oxyfluorfen on freshwater species evaluating parameters, such as growth inhibition, photosynthetic and enzymatic activities, and fatty acids profile.

Concentration/Duration	Species	Effects	References
0–20 µg/mL (72 h)	*Nostoc muscorum* *Phormidium * *foveolarum*	Significant inhibition of the photosynthetic pigments concentrations (chlorophyll a, carotenoids, and phycocyanin) and photosynthetic activity at all treatments; Significant inhibition of the photosynthetic electrons transport activity (PS II and whole chain) to both treatments; Significant growth inhibition; Significant decrease in the ability of NO_3_^−^ and PO_4_^3−^ fixation; Significant reduction of the acid phosphatase, alkaline phosphatase, and nitrate reductase to both treatments, except on *P. foveolarum,* which reported a significant increase in the nitrate reductase activity to both treatments. Significant induction in the superoxide radicals and hydrogen peroxide levels; Changes in the antioxidant enzymatic activity (catalase—CAT, superoxide dismutase—SOD, and peroxidase—POD).	[19]
0–30 µg/L (24 h)	*Scenedesmus obliquos*	Significant growth inhibition at 20 µg/L, with IC50 = 15 µg/L; Significant induction of the enzymatic activity: Glutathione reductase up to 53% at 22.5 µg/L; Glutathione *S*-transferase until 76% at 22.5 µg/L; Ascorbate peroxidase up to 29% at 22.5 µg/L; Catalase until 96% at the same concentration (22.5 µg/L).	[20]
4.3 mg/L (6 days)—acute bioassay 1.43 mg/L (15 days)—sub-acute bioassay 0.43 mg/L (30 days)—chronic bioassay	*Gambusia affinis*	Significant inhibition of acetylcholinesterase (AChE) activity (36.7%—2 days and 13.2%—6 days); Significant inhibition of AChE activity between 15.7% (5 days) and 30.64% (15 days); Significant inhibition of AChE activity between 24.5% (10 days) and 25.17% (20 days); Non-significant inhibition of AChE activity at the end of 30 days (decrease of 20.22%).	[21]
0–0.6 mg/L (21 days)	*Oreochromis * *niloticus*	Significant increase in the liver total protein content at the lower concentration (0.3 mg/L), with no significant changes at 0.6 mg/L; Significant increase in the CAT activity to both treatments at 7 and 14 days, and at 21 days to the lower concentration; Opposite trend of SOD activity that showed a significant activity inhibition for both treatments on the different days; Significant induction of GR activity; Significant induction of GST activity at 7 days, followed by a significant inhibition at 14 and 21 days; Changes in the fatty acids’ profiles to both treatments, with the most abundant being C16:0 and C18:0 (saturated fatty acids) and C18:1 and C24:1 (unsaturated fatty acids).	[22]
3 mg/L (6 days) 1mg/L (15 days) 0.3 mg/L (30 days)	*Oreochromis * *niloticus*	Significant inhibition of AChE activity up to 54.5% (2 days); Significant inhibition of AChE activity between 52.7% (5 days) and 81.28% (15 days); Significant inhibition of AChE activity between 19.7% (10 days), 54.48% (20 days), and 65.9% (30 days). Induction of the stress protein family Hsp70.	[21] [23]

**Table 3:** Effects of copper under the form of copper sulphate on several species, to evaluate different parameters, such as lethality, behavior, or biochemical indicators.

Concentration/Duration	Species/Community	Effects	References
0.01–1.00% (*w*/*v*)	*Rhodococcus erytropolis*	Effects on the bacterial cytoplasmic membrane; Decrease in the percentage of adapted cells with polarized membranes; Alteration to the fatty acids profile—increase in the saturated fatty acids and decrease in monounsaturated and polyunsaturated fatty acids.	[45]
30.2–603.4 mg/kg of wet sediment (10 days)	Microbial community of marine sediment	Effects on the biomass and metabolic activities of bacteria associated with the sediment; Changes in the community structure; Effects on activity and survival of marine metazoan fauna; Impacts on the bioturbation ability; Effects on fatty acids profile—Decrease in SFA (C14:0, C15:0, C16:0, C17:0, C18:0), MUFA (C16:1n5), and PUFA (C20:4n5, 8, 11, 14; C20:5n3) at 30 mg/kg; increase in SFA (C15:0, C17:0, C19:0) and MUFA (C17:1n7, C18:1n7) at 90 mg/kg	[41]
180–840 µg/L (96 h)	*Chaetoceros calcitrans*	Decrease in the growth rate, with the increase in the concentration and exposure time; Loss of the intact structure at 840 µg/L; Significant changes in the chlorophyll a content (increase at 180 µg/L—96 h, and decrease at 530 µg/L (75%) and at 840 µg/L (94%)) Changes in the enzymatic activity of catalase and superoxide dismutase, establishing a positive correlation with the concentrations 50, 180, and 450 µg/L.	[38]
5 and 10 µg/L (24 h)	*Phaeodactylum tricomutum * *Rhodomonas salina * *Cylindrotheca closterium*	Lipid peroxidation: Significant increase in the MDA concentration at 10 µg/L (*p* < 0.05). Significant increase in the catalase activity (*p* < 0.05), with the difference being dependent on the concentrations. Significant reduction in glutathione peroxidase activity to all treatments (*p* < 0.05). Significant increase in CAT activity at 5 µg/L. Significant increase in GPx activity at the lower concentration Significant inhibition of ascorbate peroxidase activity to all treatments. Increase in the superoxide dismutase activity, but non-significant.	[48]
200 ppb	*Gracilaria tenuistipitata*	Effects in photosynthesis process; Induction of oxidative stress; Changes in fatty acids profile—increase in SFA (C14:0, C16:0, C18:0) and MUFA (C18:1n7, C18:1n9) and decrease in PUFA (C18:2n6, C18:3n6, C18:5n4, C20:4n6, C20:5n3, 22:6n3).	[35]
Sub-lethal concentrations (72 h)	*Lamellidens marginalis*	Changes in carbohydrates—increase in lactate levels and decrease in glycogen and pyruvate levels on every treatment; Inhibition of the oxidative metabolism on the tissues—decrease in the succinate dehydrogenase and malate dehydrogenase activities; increase in the glucose-*6*-pehosphate dehydrogenase activity.	[52]
	Phototrophic organisms and macroinvertebrates	Decrease in photosynthetic health—copper’s disruptive influence on the electrons-carrying system on photosystem II [231,232]; Restructuration effect on biofilms chemical composition—dangerous effects on biofilms function [233].	[50]
0.00–2.10 mg/L (96 h) 0.00–4.00 mg/L (96 h)	*Cerastoderma edule * *Scrobicularia plana*	Large Size LC50 = 0.818 (0.595–0.987) mg/L Increase in SFA and PUFA until 31.78% and 16.60%, respectively; Decrease in MUFA and HUFA until 4.65% and 37.73%, respectively. Small Size LC50 = 1.129 (0.968–1.289) mg/L Decrease in SFA, MUFA, and PUFA until 15.43%, 11.71%, and 4.69%, respectively; Increase in HUFA up to 31.82%. Large Size LC50 = 2.563 (2.229–2.903) mg/L Increase in SFA up to 16.98% and maintenance of the levels of unsaturated fatty acids. Small Size LC50 = 4.705 (3.540–12.292) mg/L Decrease in SFA, MUFA, and PUFA until 27.14%, 13%, and 4.69%. Increase up to 24.94%.	[29]
0.00–2.10 mg/L (96 h) 0.00–4.00 mg/L (96 h)	*Cerastoderma edule * *Scrobicularia plana*	Large Size Biphasic response of GR and GST activity Increase in GPx and TBARS levels, indicating the possible occurrence of lipid peroxidation Small Size Decrease in GR, GST, and GPx activity, and TBARS levels Large Size Biphasic response of GR and GST activity Increase in GPx activity and TBARS levels, indicating the possible occurrence of lipid peroxidation Small Size Decrease in GR and GPx activity Maintenance of GST activity Biphasic response regarding TBARS levels and consequently lipid peroxidation occurrence	[29]
0–100 µg/L (5 days)	*Mytilus edulis*	Damage on DNA strongly dependent on copper concentration—Significant increase in % tail DNA compared with control, to all treatments and different damage levels among all treatments. Increase in the total glutathione levels on adductor muscle to all treatments, being significant to the organisms exposed at 32 and 56 µg/L. Histological abnormalities on adductor muscle—increase in the myocytes size and loss of the myocytes bundle structure. Histological changes in gills—hypoplasia (loss of cilia) to all treatments Loss of the digestive tubes definitions, likely by necrosis.	[54]
0.00–3.00 mg/L (96 h)	*Ctenopharyngodon idella*	LC50 = 1.717 (1.571–1.873) mg/L. Behavioral changes—anxiety, spasms, breathing difficulties, fast and erratic swimming, abrupt change in position and orientation. Fast opercular movement, frequent air intake, and remaining on one side before death, observed on the initial exposure stages, becoming occasional.	[43]
Lethal effects: 0.00–3.50 mg/L (96 h) Sub-lethal effects: 0.00, 0.11, 0.23 mg/L (60 days)	*Rutilus frisii*	LC50 = 2.310 (2.165–2.463) mg/L Damage on the growth parameters, such as significant differences on body weight to all treatments, specific growth rate to weight showing significant differences at 0.23 mg/L. Food conversation ratio and survival rate with significant differences at 0.23 mg/L.	[39]
68–244 µg/L (from egg fertilization for 120 h)	*Danio rerio* (embryonic stage)	Fewer functional neuromast and inability of the larvae to orient themselves Mortality, hatching inhibition, and impairment of the larvae development.	[40]
1–50 µg/L (2 h)	*Danio rerio* (larvae from 2 to 5 days)	Neuromast cell damage, apoptosis, and loss of ciliated cell markers.	[47]
0.15–2.50 mg/L (5 days)	*Clarias gariepinus* (embryonic stage)	Decrease in pigmentation (from 15% to 70%).	[234]
0.1641 ppm (from 24 h to 30 days)	*Penaeus indicus*	Increase in the lipid peroxidation products regarding the control (*p* < 0.0001). Significant increase in the catalase activity to all times except to 24 and 48 h (non-significant increase). Increase in the superoxide dismutase activity (from 24 h to 10 days) and decrease at 20 and 30 days.	[51]
0–110 µg/L -copper chloride (7 days)	*Ruditapes philippinarum*	Significant decrease in phagocytosis to all treatments. Significant decrease in superoxide dismutase activity to the organisms exposed to 60 and 110 µg/L. Significant increase in the hemocytes percentage showing positivity to cytochrome oxidase to the organisms exposed to 60 µg/L.	[49]

## Data Availability

The data that support the findings of this study are openly available in Mendeley Data at https://data.mendeley.com/datasets/gxkm49tvjk/2; doi: 10.17632/gxkm49tvjk.2.
